# Human umbilical cord mesenchymal stem cell conditioned medium attenuates renal fibrosis by reducing inflammation and epithelial-to-mesenchymal transition via the TLR4/NF-κB signaling pathway in vivo and in vitro

**DOI:** 10.1186/s13287-017-0760-6

**Published:** 2018-01-12

**Authors:** Bo Liu, Fengxia Ding, Dong Hu, Yu Zhou, Chunlan Long, Lianju Shen, Yuanyuan Zhang, Deying Zhang, Guanghui Wei

**Affiliations:** 1 Department of Urology, Children’s Hospital of Chongqing Medical University, Chongqing, 400014 China; 2 Department of Respiratory Medicine, Children’s Hospital of Chongqing Medical University, No. 136, Zhongshan 2 RD, Yuzhong District, Chongqing, 400014 China; 3 Ministry of Education Key Laboratory of Child Development and Disorders, Chongqing Key Laboratory of Children Urogenital Development and Tissue Engineering, Chongqing Key Laboratory of Pediatrics, Chongqing International Science and Technology Cooperation Center for Child Development and Disorders, Chongqing, 400014 China; 4 Wake Forest Institute for Regenerative Medicine, Wake Forest School of Medicine, Winston-Salem, NC 27101 USA

**Keywords:** Mesenchymal stem cell, Conditioned medium, Tubulointerstitial inflammation, Fibrosis

## Abstract

**Background:**

Renal fibrosis is characterized by infiltration of interstitial inflammatory cells and release of inflammatory mediators, activation and proliferation of fibroblasts, and deposition of excessive extracellular matrix (ECM). The aim of this study was to evaluate the effect of human umbilical cord-derived mesenchymal stem cell (hucMSC) conditioned medium (CM) on renal tubulointerstitial inflammation and fibrosis.

**Methods:**

Renal interstitial fibrosis was prepared in vivo using the unilateral ureteral obstruction (UUO). Rats were divided randomly into Sham group, Sham group with CM, UUO group, and UUO group with CM. The effect of hucMSC-CM on kidney injury induced by UUO was assessed by detecting kidney histopathology, serum creatinine (SCr), and blood urea nitrogen (BUN). The levels of TNF-α, IL-6, and IL-1β in serum and kidney tissues were detected by ELISA. The expression of proteins associated with fibrosis and renal inflammation was investigated using immunohistochemical staining and western blotting. The effects of hucMSC-CM on the TGF-β1-induced epithelial–mesenchymal transition (EMT) process and on inflammation in NRK-52E cells were investigated by immunofluorescent staining, ELISA, and western blotting.

**Results:**

hucMSC-CM reduced extracellular matrix deposition and inflammatory cell infiltration as well as release of inflammatory factors in UUO-induced renal fibrosis. Furthermore, hucMSC-CM markedly attenuated the EMT process and proinflammatory cytokines in rats with UUO and TGF-β1-induced NRK-52E cells. hucMSC-CM also inhibited the TLR4/NF-κB signaling pathway in vivo and in vitro.

**Conclusions:**

Our results suggest that hucMSC-CM has protective effects against UUO-induced renal fibrosis and that hucMSC-CM exhibits its anti-inflammatory effects through inhibiting TLR4/NF-κB signaling pathway activation.

**Electronic supplementary material:**

The online version of this article (doi:10.1186/s13287-017-0760-6) contains supplementary material, which is available to authorized users.

## Background

Chronic kidney disease (CKD) is a major public health problem affecting billions of individuals worldwide [1, 2]. At present, treatment is mainly concentrated in hemodialysis and kidney transplantation. The former faces financial constraints, while kidney transplantation is limited by donor deficiencies [3]. Therefore, it is important to elucidate the underlying pathogenesis to delay the progression of CKD and to seek effective interventions. When the kidneys are damaged, almost all types of cells including mesangial cells, endothelial cells, podocytes, renal tubular cells, and interstitial fibroblasts are involved. These cells can promote damage repair and the production of extracellular matrix [4]. At the same time, mononuclear cells, macrophages, lymphocytes, and other inflammatory cells are also involved in injury repair through different pathways [5].

Renal interstitial fibrosis is an inevitable pathological change in the development of CKD to end-stage renal disease (ESRD). Renal interstitial fibrosis is characterized by renal tubular dilation or atrophy, interstitial inflammatory cell infiltration, fibroblast proliferation, and increased interstitial matrix deposits [6]. Interleukin, monocyte chemotactic protein 1 (MCP-1) involved in the process of renal interstitial fibrosis, and the release of local inflammatory factors also increased renal interstitial fibrosis [7]. At present, there is no special treatment for renal interstitial fibrosis. Therefore, it is imperative to find appropriate treatment to delay the progress of renal interstitial fibrosis.

Recent studies on unilateral ureteral obstruction (UUO) [8], glycerol [9], and platinum-induced kidney injury [10] have shown that mesenchymal stem cells (MSCs) have the effect of inhibiting renal tubular epithelial cell apoptosis, promoting renal tubular epithelial cell proliferation via a paracrine mechanisms, or directly differentiating into intrinsic renal cells for repair. In addition to directly promoting the repair of damaged tissue, MSCs also showed an immune system modulating effect and improved tissue damage caused by excessive inflammation. The reason may be that MSCs can secrete many different kinds of cytokines and growth factors, and these factors have anti-inflammatory, immune regulation, inhibition of apoptosis, and stimulating regeneration effects [11]. Recent studies have shown that infusion of MSC conditioned medium can effectively improve cisplatin-induced acute kidney injury and further confirm that MSCs play a protective role by paracrine secretion [12]. Until now, the protective effect of human umbilical cord-derived mesenchymal stem cell (hucMSC) conditioned medium (CM) on renal fibrosis has not been evaluated. Therefore, our study evaluated the anti-inflammatory effect of hucMSC-CM in CKD rats and elucidated its underlying mechanism.

## Methods

### Ethics statement

The study involving both human and animals was conducted in accordance with the principles of the Helsinki Declaration and was approved by the ethical committee of Chongqing Medical University (File No. 2016-124).

### Isolation, expansion, and characteristics of hucMSCs

After obtaining parental and ethics committee consent, hucMSCs were isolated as described previously [13]. The cells were cultured in Dulbecco’s modified Eagle’s medium nutrient mixture F-12 (DMEM/F12) with 10% fetal bovine serum (FBS) and 1% penicillin/streptomycin (Gibco, USA) at 37 °C with 5% CO_2_. The media were subsequently exchanged every 3 days. The phycoerythrin (PE)-conjugated antibodies were CD34, CD45, CD73, CD90, CD105, and human leukocyte antigen (HLA)-DR (BD Biosciences). The multilineage differentiation of hucMSCs was determined using adipogenic and osteogenic medium (Cyagen, China). Briefly, a total of 1 × 10^5^ hucMSCs at passage 2 were seeded in a six-well plate and then incubated with induced medium. After 2 weeks, adipogenic differentiation of hucMSCs was observed by Oil Red O staining, and osteogenic differentiation of hucMSCs was examined by Alizarin Red staining (Additional file 1: Figure S1).

### Collection and concentration of hucMSC-CM

Passage 3 hucMSCs were cultured to 80–90% confluence in a T75 culture flask (4 × 10^6^ cells). After the cells were washed three times with phosphate-buffered saline (PBS), the serum-free DMEM medium was refilled (10 ml) and further incubated for 48 hours. The collected medium was centrifuged for 10 min at 3000 rpm in order to remove cell debris. CM was concentrated 25 times using 10 kDa MW cutoff filter units (Millipore) and sterilized by filtration through a 0.22-μm filter. After these steps, the mean protein concentration of the concentrated CM is about 0.5 mg/ml. All of the concentrated hucMSC-CM was stored at –80 °C until use.

### In-vitro experimental treatment of NRK-52E

NRK-52E cells were purchased from China Center for Type Culture Collection (CCTCC), and cultured in DMEM medium containing 10% FBS (Gibco, Grand Island, NY, USA) and 2% penicillin/streptomycin at 37 °C with 5% CO_2_. NRK-52E cells with good morphology were digested and seeded in six-well plates at a cell concentration of about 1 × 10^5^ cells per well. After the cells attached, recombinant human transforming growth factor β1 (TGF-β1) (R&D, Minneapolis, MN, USA) (5 ng/ml) was added to induce apoptosis with hucMSC-CM (40 μg) treatment. After 48 hours, the cell slide was used for immunofluorescent staining, and the proteins in cells were extracted for western blot assay.

### UUO rat model and injection of hucMSC-CM

In this study, male SD rats with weight average 200 ± 10 g were provided and raised at Chongqing Medical University (SPF, License No.: SYXK (Chongqing) 2007-0001). The rats were raised in polycarbonate cages in a human-controlled environment, such as free water and food, with an illumination schedule of 12-hour light/12-hour dark.

The rats were categorized randomly into four groups (*n* = 8 in each group), including PBS transplanted with sham operation (Sham group), CM transplanted with sham operation (Control group), PBS transplanted with UUO (UUO group), and CM transplanted with UUO (hucMSC-CM group). The male SD rats were anesthetized with chloral hydrate. The left ureter and kidney were then exposed with the assistance of flank incision; the ligation process of the left ureter was conducted with two 4-0 silk sutures at the upper third of the ureter, then cut between the ligatures to prevent retrograde urinary tract infection. Sham-treated rats underwent an identical procedure except for ureteral ligation. The Control and hucMSC-CM groups received hucMSC-CM (100 μl) via the left renal artery after the surgery. All groups received antibiotics (0.1% amoxicillin) in their drinking water for 14 days. At days 14 post surgery, the rats were euthanized and the left kidneys were extracted. One portion of each kidney was fixed in paraformaldehyde solution (4%), and the other portions were stored in liquid nitrogen. Blood samples were collected in order to measure biochemical analysis, including serum creatinine (SCr) and blood urea nitrogen (BUN).

### Histological examination

In order to explore the degree of renal tubular injury and renal interstitial fibrosis, the kidney was fixed with 4% paraformaldehyde, embedded in paraffin, and cut to 4 μm thick. The sections were dewaxed with xylene and then incubated sequentially for 5 min in different concentrations of alcohol and water before hematoxylin–eosin (HE), periodic acid–Schiff (PAS), and Masson’s trichrome staining. The damaged degree of tubular cells was scored and divided into four levels: 0 = normal without any damages; 1 = mild dilatation; 2 = flattened epithelial cells and loss of brush border; and 3 = denudation of basement membranes, tubular cell apoptosis and necrosis. The total score is calculated as the average of all tubular scores with 200 tubuli per section. Ten visual fields were selected randomly in digital images from each section under 40× magnification and then the percentage of fibrotic areas to overall field areas was calculated.

### Measurement of TNF-α, IL-6, IL-1β, and MCP-1

In order to determine the levels of TNF-α, IL-6, and IL-1β in blood serum and kidney tissues of each group, ELISA kits from R&D Systems were used and operated according to the manufacturer’s instructions. The levels of TNF-α and MCP-1 (BIOSOURCE, USA) in the cell supernatant were measured by the same method.

### Immunofluorescent and immunohistochemistry

NRK-52E cells were treated as already described, fixed with 4% paraformaldehyde, and permeabilized with Triton X100 (Solarbio, China). The cell slides were then blocked with 5% BSA (BD, USA) and incubated with cytokeratin 8 (1:100; Abcam, USA), E-cadherin (1:200; Abcam, USA), α-SMA (1:200; Abcam, USA), TLR4 (1:200; Santa Cruz, USA), and p-NF-κB (1:200; CST, USA) at 4 °C overnight. After washing three times with PBS, the corresponding fluorescent secondary antibody (ZSGB-BIO, China) was added to incubate for 1 hour. After nuclear counterstaining with DAPI (Beyotime, China), images were examined under a fluorescence microscope (K10587; Nikon, Japan).

The kidney tissue slices were underwent dewaxing and hydration, and antigen was retrieved by citric acid buffer (PH 6.0) microwave antigen retrieval. After natural cooling, the endogenous peroxidase was blocked with 3% H_2_O_2_ for 10 min, and then the nonspecific protein binding site was blocked with 0.5% BSA for 1 hour. Slices were incubated at 4 °C with 1:200 diluted mouse anti-E-cadherin antibody (Santa Cruz, USA), rabbit anti-Collagen-1 antibody (Abcam, USA), mouse anti-α-smooth muscle actin antibody (Abcam, USA), rabbit anti-CD3 antibody (Abcam, USA), rabbit anti-CD68 antibody (Abcam, USA), rabbit anti-MCP-1 antibody (Abcam, USA), rabbit anti-TNF-α (Genetex, USA), mouse anti-TLR-4 antibody (Santa Cruz, USA), and rabbit anti-phosphorylated-NF-κB antibody (CST, USA), respectively. Slices were washed three times with PBS, then with the goat anti-rabbit or anti-mouse antibody (Zhongshan, Beijing, China) at 37 °C for 1 hour. After sections were counterstained with hematoxylin, positive staining was developed by incubation with 3,3′-diamnobenzidine (DAB). The intensity of E-cadherin, Collagen-I, α-SMA, MCP-1, TNF-α, TLR-4, and phosphorylated-NF-κB was assessed under five randomly selected high-power fields (×400). Image Pro Plus 6.0 was used as a tool to quantify the integrated optical density (IOD) of positive areas.

### Western blot analysis

Kidney tissues and cells were lysed in RIPA Lysis Buffer (Beyotime, China) containing PMSF, and centrifuged at 4 °C at 12,000 rpm for 20 min. The protein concentration was measured using the BCA assay kit (Beyotime, China). Sodium dodecyl sulfate polyacrylamide gel electrophoresis (SDS-PAGE) loading buffer was mixed with protein samples and boiled last for 10 min. The proteins were electrophoresed through SDS-polyacrylamide gels. The proteins were then transferred to polyvinylidene fluoride (PVDF) membranes (Millipore, USA). In order to block the nonspecific protein background, membranes were blocked in 5% skim milk for 1 hour with a shaker. After that, the protein bands were incubated overnight at 4 °C with the following primary antibodies: mouse anti-E-cadherin (1:1000; Santa Cruz, USA), rabbit anti-Collagen-I (1:1000; Abcam, USA), mouse anti-α-SMA (1:1000; Abcam, USA), mouse anti-cytokeratin 8 (1:1000; Abcam, USA), rabbit anti-CD68 (1:500; Abcam, USA), rabbit anti-MCP-1 (1:2000; Abcam, USA), rabbit anti-TNF-α (1:1000; Genetex, USA), mouse anti-TLR-4 (1:1000; Santa Cruz, USA), rabbit anti-NF-κB (1:1000; Proteintech, USA), rabbit anti-phosphorylated-NF-κB (1:1000; CST, USA), rabbit anti-IκBα (1:400; Bioss, China), rabbit anti-phosphorylated-IκBα (1:400; Bioss, China) and β-actin (1:500; Zhongshan, China). The membrane was then washed three times with Tris-buffered saline/Tween (TBST) and incubated in goat anti-rabbit or mouse antibodies (Zhongshan, China) for 2 hours at 37 °C. Immobilon Western Chemiluminescent HRP Substrate (Millipore, USA) were used to detect positive immune reactions.

### Statistical analysis

The data obtained from the experiment were expressed as mean ± standard deviation (SD). The mean value of each group was compared with one-way analysis of variance (ANOVA) followed by Dunnett’s post-hoc test. When *P* < 0.05, the difference was considered statistically significant. All data were analyzed by SPSS 16.0 software.

## Results

### hucMSC-CM improved the pathological structure and function of UUO-induced kidney in rats

**Fig. 1:**
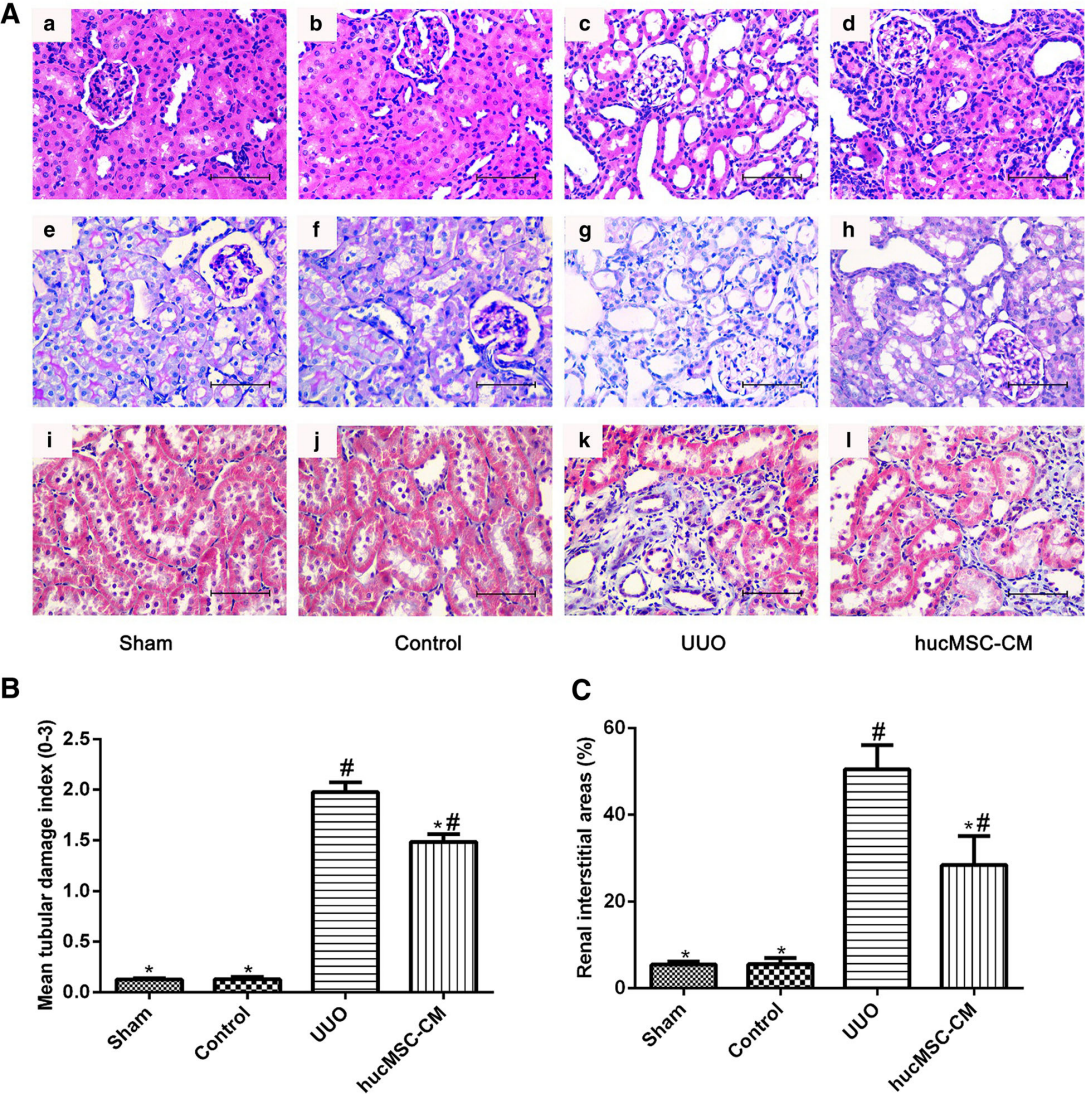
**A** Histomorphological analysis of the kidney in each group 14 days postoperatively. (a–d) H&E staining. (e–h) PAS staining. (i–l) Masson’s trichrome staining. **B** Results of renal tubular histology score. Loss of brush border, tubular dilation, and apoptosis/necrosis of tubular cells were assessed. **C** Area of renal interstitial fibrosis in each group. hucMSC-CM transplantation could significantly inhibit renal interstitial fibrosis in UUO kidney. Values presented as mean ± SD. UUO group compared with Sham, Control, and hucMSC-CM groups. Values statistically significant at: *P < 0.05, Sham and Control groups compared with UUO and hucMSC-CM groups; ^#^*P* < 0.05. CM conditioned medium, hucMSC human umbilical cord-derived mesenchymal stem cell, UUO unilateral ureteral obstruction

**Fig. 2:**
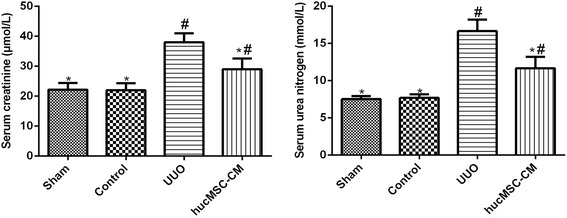
Biochemical analysis of the kidney in each group 14 days postoperatively. Levels of serum creatinine (SCr) and blood urea nitrogen (BUN) detected. hucMSC-CM inhibited the increase of SCr and BUN in UUO-induced rats. Values presented as mean ± SD. UUO group compared with Sham, Control, and hucMSC-CM groups. Values statistically significant at: *P < 0.05, Sham and Control groups compared with UUO and hucMSC-CM groups; ^#^*P* < 0.05. CM conditioned medium, hucMSC human umbilical cord-derived mesenchymal stem cell, UUO unilateral ureteral obstruction

As shown in Fig. 1, there was no significant pathological change in the glomeruli and tubules in the Sham and Control groups. Fourteen days postoperatively, inflammatory cell infiltration, renal tubular dilatation, atrophy or necrosis, and interstitial fibrosis can be detected easily. In addition, PAS staining was used to assess the number and degree of damaged renal tubules; the proximal and distal tubules were intact in the Sham and Control groups 14 days postoperatively, but marked disorganization of the structure of proximal and distal tubules with brush border disruption could be seen in the UUO group. However, the number and the degree of damaged renal tubules in the hucMSC-CM group were less than that in the UUO group (*P* < 0.05). Masson’s trichrome staining showed that few collagens were deposited around renal tubules in the Sham and Control groups 14 days postoperatively. However, the collagen fiber was deposited in the renal interstitium, and interstitial fibrosis can be detected easily in the UUO group. Furthermore, the interstitial fibrosis area of the hucMSC-CM group was significantly lower than that of the UUO group (*P* < 0.05). We also examined the levels of serum creatinine (SCr) and blood urea nitrogen (BUN) after UUO surgery (Fig. 2). We found that SCr and BUN levels were significantly increased at 14 days after UUO, and SCr and BUN levels were significantly decreased after hucMSC-CM treatment (*P* < 0.05).

### hucMSC-CM inhibited UUO-induced TNF-α, IL-1β, and IL-6 production

**Fig. 3:**
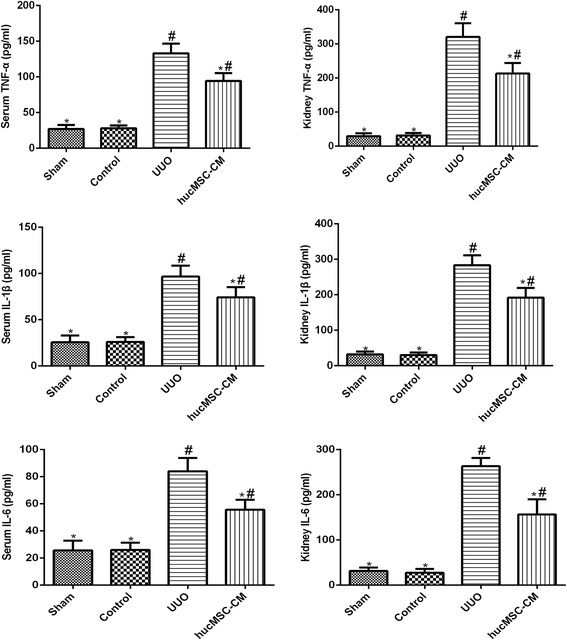
Effects of hucMSC-CM on UUO-induced TNF-α, IL-1β, and IL-6 in serum and kidney tissues. hucMSC-CM inhibited the increase of TNF-α, IL-1β, and IL-6 in UUO-induced rats. Values presented as mean ± SD. UUO group compared with Sham, Control, and hucMSC-CM groups. Values statistically significant at: *P < 0.05, Sham and Control groups compared with UUO and hucMSC-CM groups; ^#^*P* < 0.05. CM conditioned medium, hucMSC human umbilical cord-derived mesenchymal stem cell, IL interleukin, TNF tumor necrosis factor, UUO unilateral ureteral obstruction

The effect of hucMSC-CM on the production of inflammatory cytokines TNF-α, IL-1β, and IL-6 was examined in order to study the anti-inflammatory effect of hucMSC-CM on UUO-induced CKD. The results showed that compared with the Control and Sham groups, the production of TNF-α, IL-1β, and IL-6 in serum and kidney tissues was significantly increased in the UUO group. However, hucMSC-CM inhibited UUO-induced TNF-α, IL-1β, and IL-6 production (Fig. 3).

### hucMSC-CM inhibited epithelial–mesenchymal transition in UUO-induced rats

**Fig. 4:**
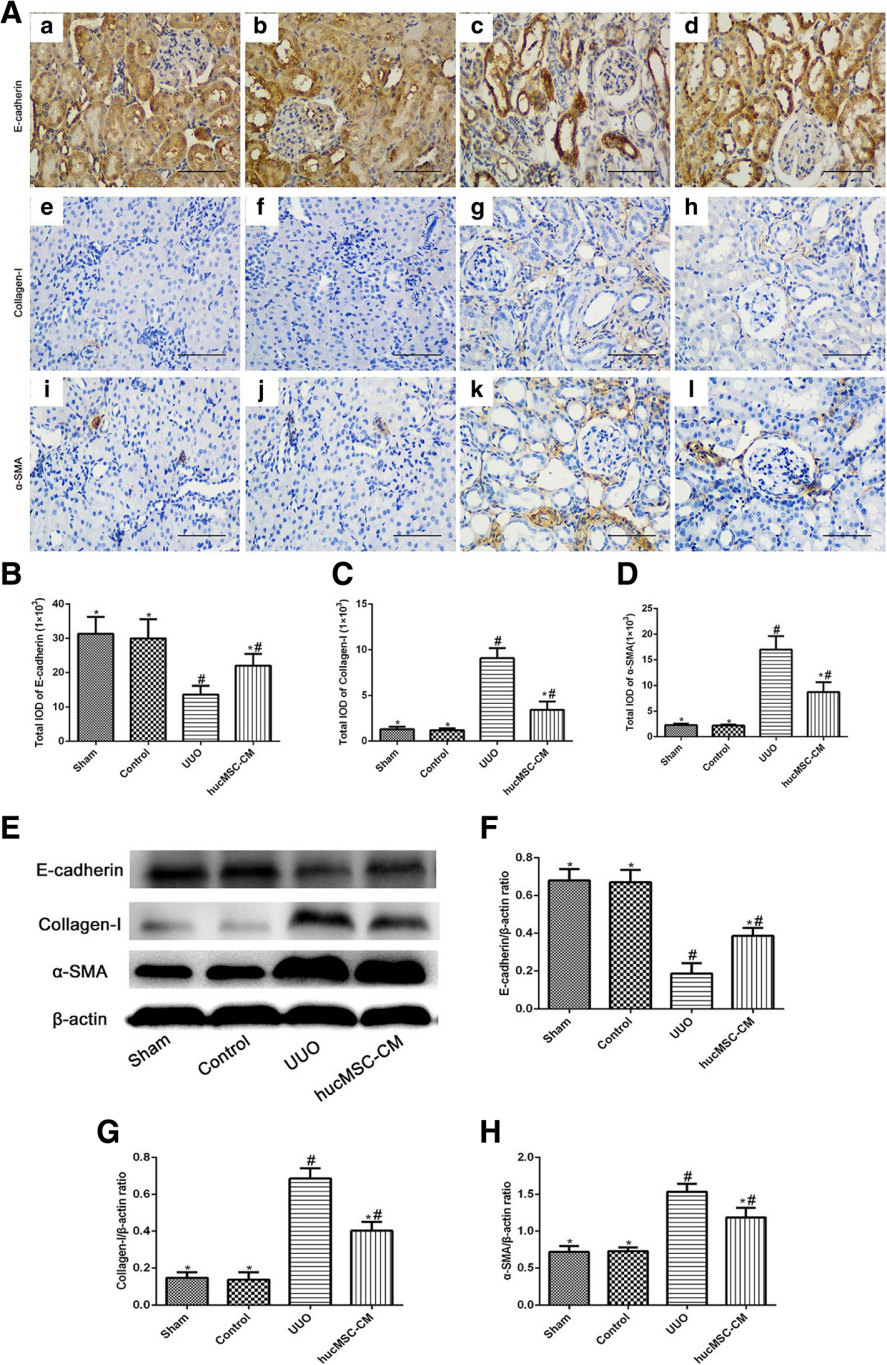
Effects of hucMSC-CM on expression of E-cadherin, Collagen-I, and α-SMA in each group 14 days postoperatively. **A** Immunohistochemical staining of kidney tissue sections using E-cadherin (a–d), Collagen-I (e–h), and α-SMA (i–l). **B**–**D** Total IOD for E-cadherin, Collagen-I, and α-SMA in each group. **E** Western blot analysis of E-cadherin, Collagen-I, and α-SMA protein in each group. **F**–**H** Relative protein expression levels in kidney tissues. β-actin used as inner reference and quantified using densitometric analysis. Values presented as mean ± SD. UUO group compared with Sham, Control, and hucMSC-CM groups. Values statistically significant at: *P < 0.05, Sham and Control groups compared with UUO and hucMSC-CM groups; ^#^*P* < 0.05. CM conditioned medium, hucMSC human umbilical cord-derived mesenchymal stem cell, IOD integrated optical density, SMA smooth muscle actin, UUO unilateral ureteral obstruction

Epithelial–mesenchymal transition (EMT) is an important mechanism of renal interstitial fibrosis [6]. Therefore, we examined the important indicators of EMT in the kidney of CKD. Through the immunohistochemical staining and western blot analysis of the related proteins, we found that the expression of E-cadherin (renal tubular epithelial marker) decreased after UUO, while the expression of α-SMA (fibroblast marker) was significantly increased, and the expression of collagen-I in renal interstitium was also significantly increased. Compared with UUO rats, hucMSC-CM could significantly inhibit the expression of Collagen-I and α-SMA in the kidney of UUO rats and promote the expression of E-cadherin. These results suggest the potential role of hucMSC-CM in inhibiting renal interstitial fibrosis in CKD (Fig. 4).

### hucMSC-CM improved interstitial inflammation in UUO-induced rats

**Fig. 5:**
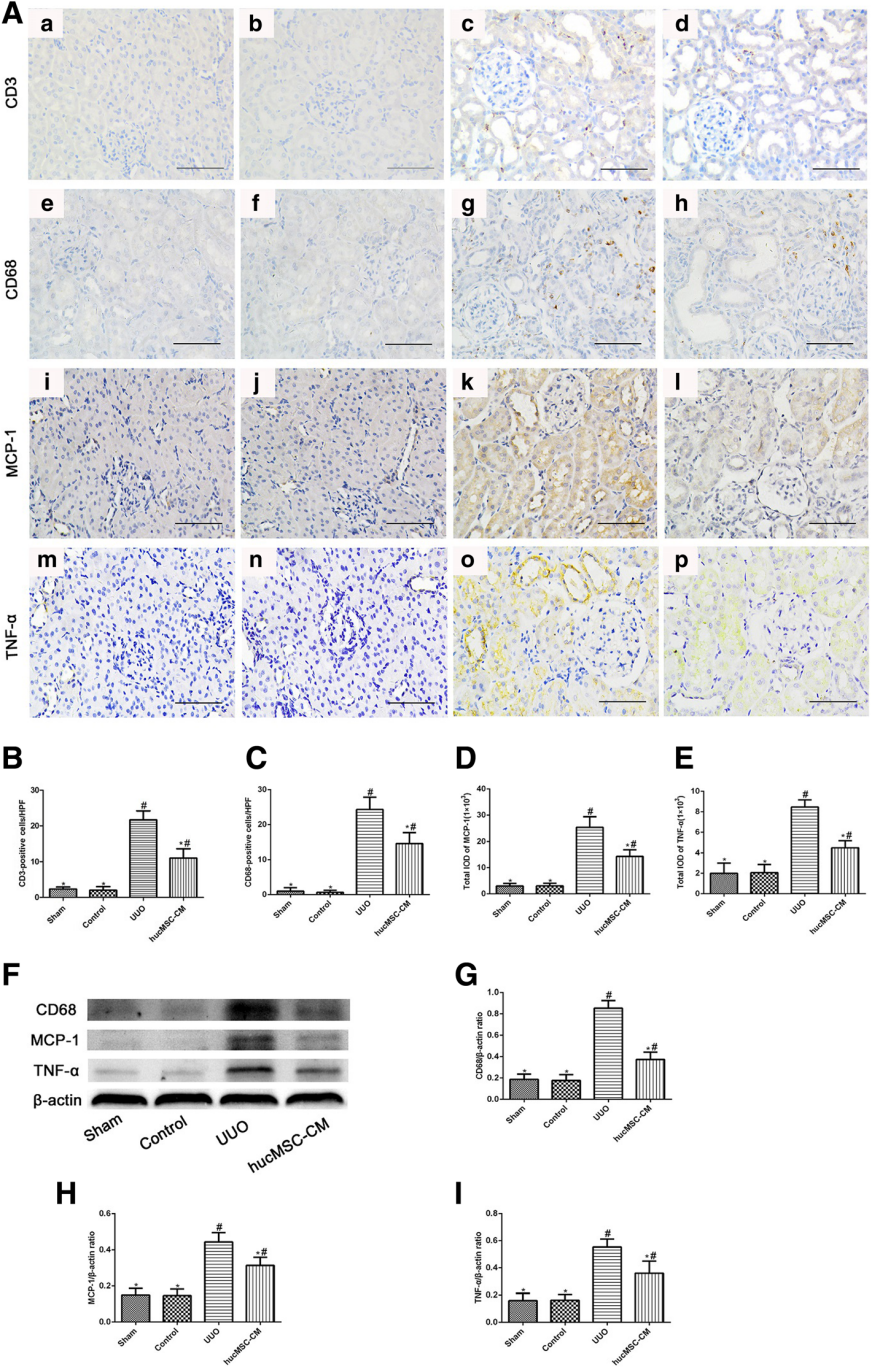
Effects of hucMSC-CM on expression of CD3, CD68, MCP-1, and TNF-α in each group 14 days postoperatively. **A** Immunohistochemical staining of kidney tissue sections using CD3 (a–d), CD68 (e–h), MCP-1 (i–l), and TNF-α (m–p). **B**–**E** CD3-positive and CD68-positive cells under high-power field (HPF) and total IOD for MCP-1 and TNF-α in each group. **F** Western blot analysis of CD68, MCP-1, and TNF-α protein in each group. **G**–**I** Relative protein expression levels in kidney tissues. β-actin used as inner reference and quantified using densitometric analysis. Values presented as mean ± SD. UUO group compared with Sham, Control, and hucMSC-CM groups. Values statistically significant at: *P < 0.05, Sham and Control groups compared with UUO and hucMSC-CM groups; ^#^*P* < 0.05. CM conditioned medium, hucMSC human umbilical cord-derived mesenchymal stem cell, IOD integrated optical density, MCP-1 monocyte chemotactic protein 1, TNF tumor necorsis factor, UUO unilateral ureteral obstruction

Because renal interstitial fibrosis is manifested initially as an interstitial inflammatory response and eventually leads to extensive fibrosis changes [14], we identified several inflammatory markers by immunohistochemical staining and western blotting including CD3 (lymphocyte marker), CD68 (macrophage marker), monocyte chemotactic protein-1 (MCP-1), and tumor necrosis factor-α (TNF-α). Our results suggested that hucMSC-CM treatment significantly reduced lymphocyte and macrophage infiltration in renal interstitium compared with UUO-induced rats, and the expression of MCP-1 and TNF-α was also significantly reduced (Fig. 5).

### hucMSC-CM inhibited activation of the TLR4/NF-κB signaling pathway in UUO-induced rats

**Fig. 6:**
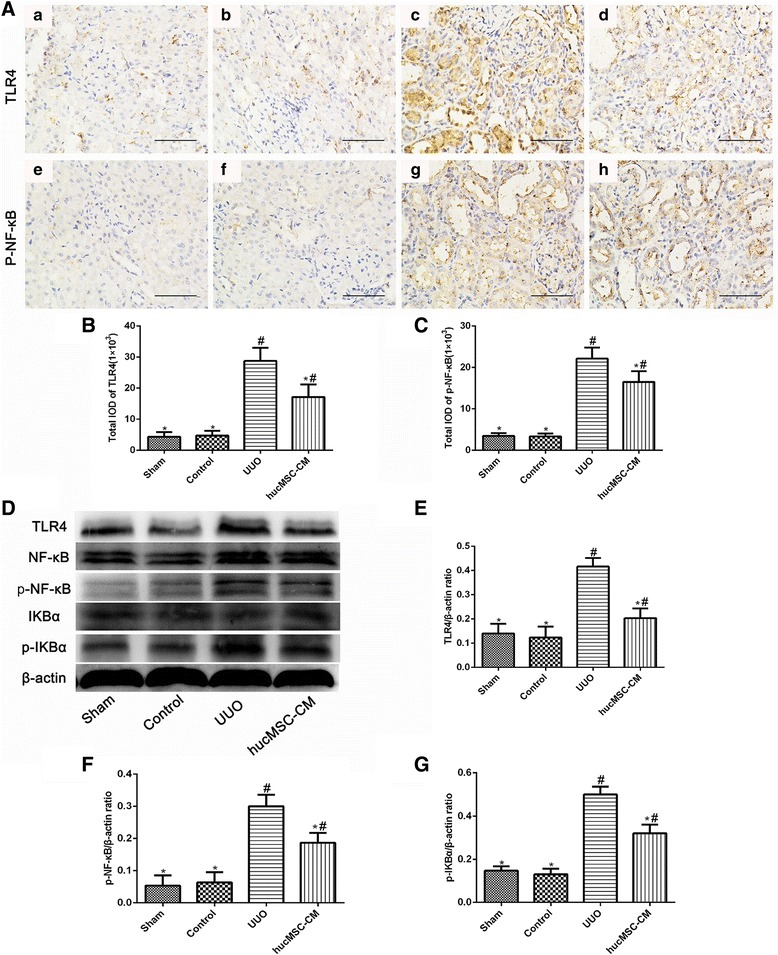
Effects of hucMSC-CM on expression of TLR4, NF-κB, and IκBα in each group 14 days postoperatively. **A** Immunohistochemical staining of kidney tissue sections using TLR4 (a–d) and p-NF-κB (e–h). **B**, **C** Total IOD for TLR4 and p-NF-κB in each group. **D** Western blot analysis of TLR4, NF-κB, p-NF-κB, IκBα, and p-IκBα protein in each group. **E**–**G** Relative protein expression levels in kidney tissues. β-actin used as inner reference and quantified using densitometric analysis. Values presented as mean ± SD. UUO group compared with Sham, Control, and hucMSC-CM groups. Values statistically significant at: *P < 0.05, Sham and Control groups compared with UUO and hucMSC-CM groups; ^#^*P* < 0.05. CM conditioned medium, hucMSC human umbilical cord-derived mesenchymal stem cell, IOD integrated optical density, p-NF-κB phosphorylated nuclear factor-κB, TLR4 Toll-like receptor 4, UUO unilateral ureteral obstruction

In order to investigate the anti-inflammatory mechanism of hucMSC-CM, the effect of hucMSC-CM on UUO-induced activation of the Toll-like receptor 4 (TLR4)/NF-κB signaling pathway was examined. As shown in Fig. 6, the results of immunohistochemical staining and western blot analysis showed that hucMSC-CM significantly inhibited UUO-induced TLR4/NF-κB activation and NF-κBα inhibitory protein (IκBα) degradation in rats.

### hucMSC-CM inhibited TGF-β1-induced EMT in NRK-52E cells

**Fig. 7:**
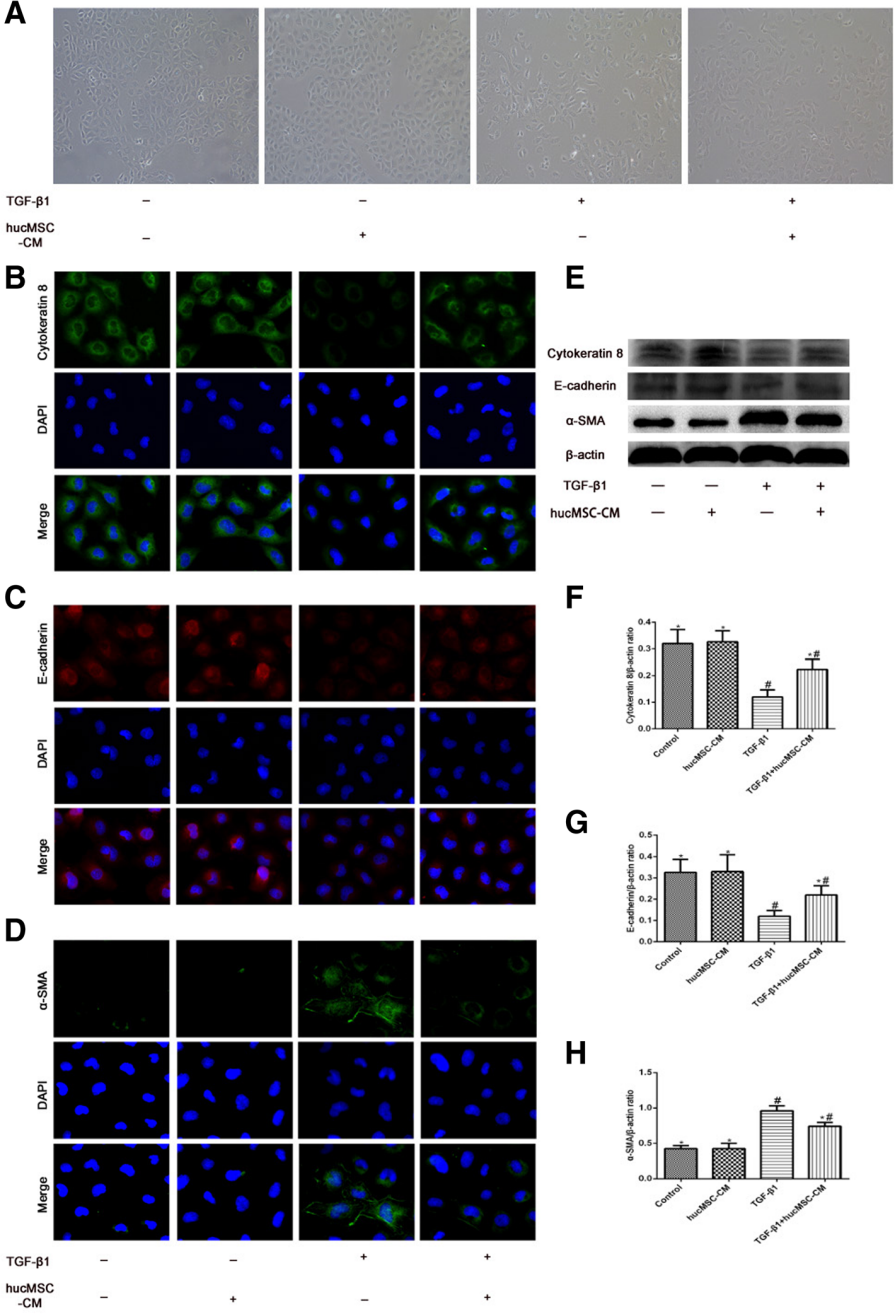
Effects of hucMSC-CM on EMT process of NRK-52E cells in each group at 48 hours. **a** Representative micrographs from each group. **b**–**d** Immunofluorescent staining for cytokeratin 8, E-cadherin, and α-SMA in each group. **e** Western blot analysis of cytokeratin 8, E-cadherin, and α-SMA protein in each group. **f**–**h** Relative protein expression levels in NRK-52E cells. β-actin used as inner reference and quantified using densitometric analysis. Values presented as mean ± SD. TGF-β1 group compared with Control and hucMSC-CM groups. Values statistically significant at: *P < 0.05, Control group compared with TGF-β1 and hucMSC-CM groups; ^#^*P* < 0.05. CM conditioned medium, DAPI 4′,6-diamidino-2-phenylindole, hucMSC human umbilical cord-derived mesenchymal stem cell, SMA smooth muscle actin, TGF-β1 transforming growth factor β1, UUO unilateral ureteral obstruction

EMT plays an important role in the development and progression of interstitial fibrosis. Therefore, we studied the changes of EMT induced by TGF-β1 in NRK-52E cells and the therapeutic effect of hucMSC-CM. As shown in Fig. 7a, NRK-52E cells in the Control group exhibited the typical cobblestone morphology of epithelial cells. After TGF-β1 treatment for 48 hours, the cells were elongated and showed fibroblast-like morphology. hucMSC-CM and TGF-β1 coincubation inhibited the phenotype of NRK-52E cell change to the myofibroblasts and restored the epithelial morphology of the NRK-52E cells. In addition, we used cell immunofluorescence staining and western blotting to detect the expression of epithelial markers (cytokeratin 8, E-cadherin) and fibroblast marker (α-SMA) during EMT. The results showed that hucMSC-CM inhibited suppression of cytokeratin 8 and E-cadherin in NRK-52E cells induced by TGF-β1, and inhibited the expression of α-SMA (Fig. 7b–h).

### hucMSC-CM attenuated TGF-β1-induced TNF-α and MCP-1 secretion in NRK-52E cells

**Fig. 8:**
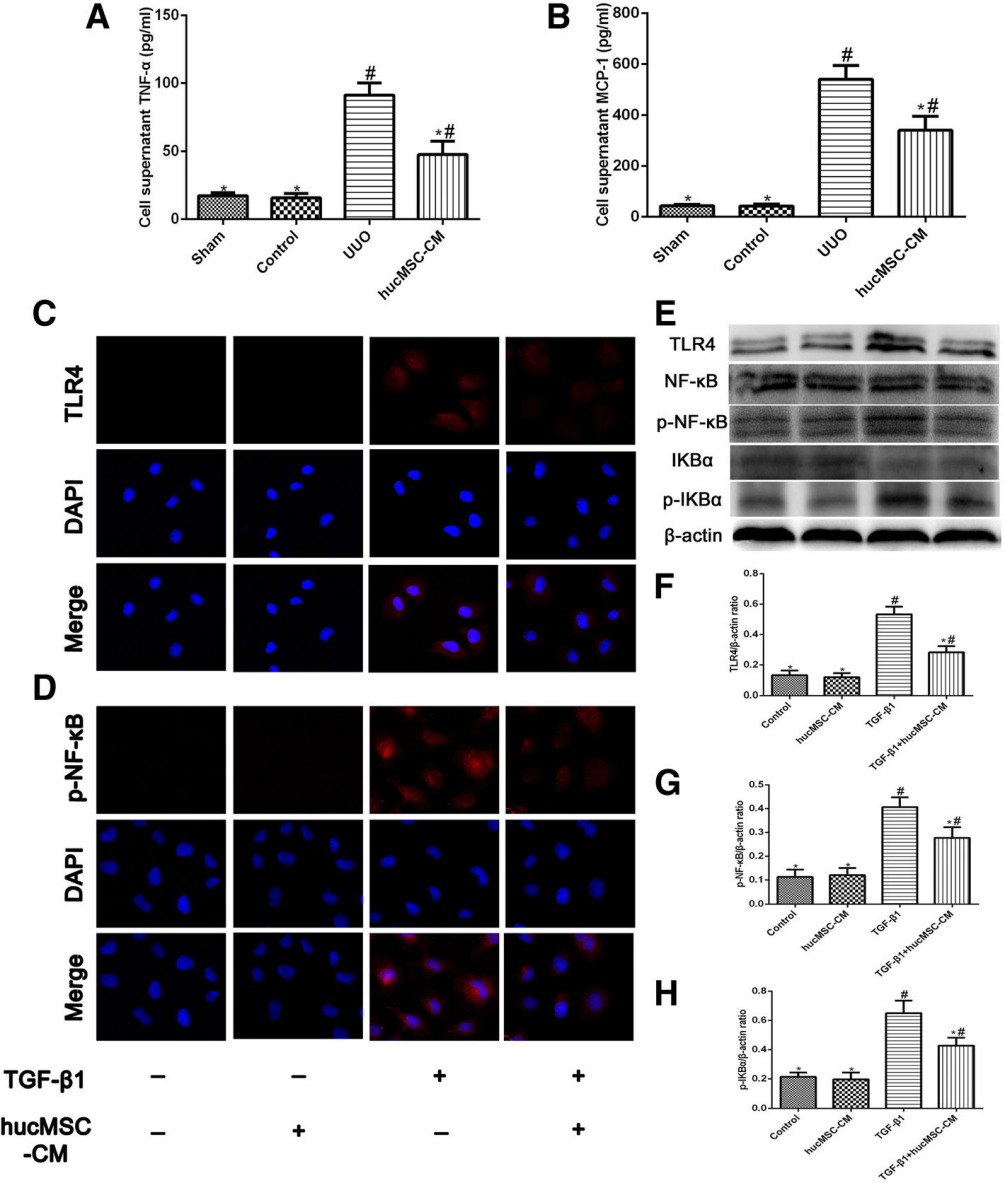
**a**, **b** Effects of hucMSC-CM on inflammatory cytokines secreted by NRK-52E in each group at 48 hours. hucMSC-CM inhibited the increase of TNF-α and MCP-1 secreted by TGF-β1-induced NRK-52E cells. **c**, **d** Immunofluorescent staining for TLR4 and p-NF-κB in each group. **e** Western blot analysis of TLR4, NF-κB, p-NF-κB, IκBα, and p-IκBα protein in each group. **f**–**h** Relative protein expression levels in NRK-52E cells. β-actin used as an inner reference and quantified using densitometric analysis. Values presented as mean ± SD. TGF-β1 group compared with Control and hucMSC-CM groups. Values statistically significant at: *P < 0.05, Control group compared with TGF-β1 and hucMSC-CM groups; ^#^*P* < 0.05. CM conditioned medium, DAPI 4′,6-diamidino-2-phenylindole hucMSC human umbilical cord-derived mesenchymal stem cell, IOD integrated optical density, MCP-1 monocyte chemotactic protein 1, p-NF-κB phosphorylated nuclear factor-κB, TGF-β1 transforming growth factor β1, TLR4 Toll-like receptor 4, TNF tumor necrosis factor, UUO unilateral ureteral obstruction

Previous studies have suggested that NF-κB signaling played an essential role in TGF-β1-induced EMT [15]. Once NF-κB signaling is activated, NRK-52E cells can be induced to express proinflammatory cytokine TNF-α and chemokine MCP-1 [16]. Further, we investigated whether hucMSC-CM is involved in the process of TGF-β1-induced inflammatory response in renal tubular epithelial cells. After TGF-β1 induced NRK-52E cells for 48 hours, the levels of TNF-α and MCP-1 in the culture medium increased significantly, and the elevated levels of both were inhibited by cocultured with hucMSC-CM (Fig. 8a, b).

### hucMSC-CM inhibited TGF-β1-induced activation of the TLR4/NF-κB signaling pathway in NRK-52E cells

Considering the important role of TLR4/NF-κB in the development of inflammation, we also examined the effect of hucMSC-CM on TGF-β1-induced changes of TLR4, NF-κB, and IκB expression. As shown in Fig. 8c–h, the results of immunofluorescence staining and western blot analysis showed that hucMSC-CM significantly inhibited TGF-β1-induced TLR4/NF-κB signaling activation and IκBα degradation in NRK-52E cells.

## Discussion

Renal fibrosis is a common final outcome of progressive CKD [17]. An important feature of renal interstitial fibrosis is the infiltration of interstitial inflammatory cells and the release of inflammatory mediators, while the activation and proliferation of fibroblasts can cause deposition of excessive extracellular matrix (ECM) [6]. Currently, there is no effective way to treat renal fibrosis. Therefore, there is an urgent need to further clarify the pathogenesis of renal fibrosis and to find novel therapeutic intervention.

In this study, the effect of hucMSC-CM on UUO-induced CKD was detected. Changes in SCr and urea nitrogen levels are markers of renal dysfunction [18]. In order to assess the effect of hucMSC-CM on the renal function of UUO model, we tested the changes of SCr and urea nitrogen in each group. The results showed that hucMSC-CM significantly inhibited UUO-induced increase of SCr and BUN levels, suggesting that hucMSC-CM can effectively improve UUO-induced renal dysfunction. Furthermore, the pathological results of the kidneys in each group showed that hucMSC-CM improved UUO-induced renal injury, and significantly improved renal tubular injury and renal interstitial fibrosis. These results suggested that hucMSC-CM had a protective effect against UUO-induced CKD in rats.

The occurrence of EMT in damaged renal tubular epithelial cells plays an important role in the progression of renal interstitial fibrosis [6]. In this process, renal tubular epithelial cells lost the phenotype of epithelial cells while obtaining the mesenchymal cell phenotype, leading to a substantial increase in the number of myofibroblasts, and ultimately leading to the occurrence of renal interstitial fibrosis [19]. Our results found that UUO can induce the occurrence of EMT in renal tubular epithelial cells, and loss of intercellular epithelial adhesion molecule E-cadherin, which is an important determinant for maintenance of the epithelial phenotype [20]. Meanwhile, UUO can induce a significant increase in the expression of α-SMA (a marker of myofibroblasts). The ultimate end was extracellular matrix deposition and renal interstitial fibrosis, showing a significant increase in the expression of collagen-I protein. TGF-β1 is an extremely potent profibrotic factor that stimulates proliferation and activation of fibroblasts and induces EMT, resulting in increased ECM synthesis and decreased degradation, and therefore in excessive deposition of ECM in the renal interstitium [19]. We also used TGF-β1 to induce rat renal tubular epithelial cells (NRK-52E) to simulate the process of EMT and evaluate the effect of hucMSC-CM in vitro. Our results suggested that hucMSC-CM inhibited the process of EMT and improved renal fibrosis both in vivo and in vitro.

Damaged tubular cells, infiltrating lymphocytes, macrophages, and accumulated fibroblasts produce cytokines and growth factors that allow UUO-induced kidneys to be in an inflammatory state. This inflammatory condition further leads to tubular atrophy and interstitial fibrosis [7]. The accumulation of macrophages and T lymphocytes in the renal interstitium is closely related to the progress of renal injury [14]. Therefore, we tested the levels of proinflammatory cytokines (IL-1, IL-6, and TNF-α) and chemokines (MCP-1) in serum, kidney tissues, and cell supernatant. In the meantime, we examined the infiltration of lymphocytes and macrophages in kidney tissue. The results confirmed that hucMSC-CM has a role in regulating the inflammatory response and reducing the production of inflammatory cells and factors, which is a potential protective mechanism of hucMSC-CM for UUO-induced CKD.

The activated TLR4/NF-κB signaling pathway is an important contributor to the inflammatory response in kidney disease [14, 21, 22]. TLR4 induces the expression of inflammatory cytokines and activates the NF-κB pathway as a promoter to stimulate proinflammatory reaction. In the normal state, NF-κB is sequestered in the cytoplasm and bound with IκB [23]. Once the pathway is active, NF-κB changes position into the nucleus to manage the production of inflammatory cytokines [24]. Previous studies have shown that activation of the TLR4/NF-κB signaling pathway can cause interstitial inflammation, which in turn leads to renal interstitial fibrosis, and inhibition of TLR4/NF-κB pathway activation can inhibit the progression of renal fibrosis [14, 25]. In this study, UUO-induced rats were used to evaluate the protective mechanism of hucMSC-CM against CKD. The results showed that hucMSC-CM inhibited NF-κB activity and p-IκB expression. Moreover, hucMSC-CM attenuated the activation of TLR4. Together with those results, we conclude that hucMSC-CM exhibited its anti-inflammatory effects by inhibiting activation of the TLR4/NF-κB signaling pathway, thereby inhibiting the EMT process and improving renal fibrosis.

Although our results have suggested that hucMSC-CM can reduce the expression of inflammatory cytokines in CKD, the specific route of hucMSC regulation of immune response and the local inflammatory microenvironment is not completely understood. Previous studies suggested that the paracrine effect of hucMSCs plays an important role in trauma repair and regulation of inflammation response. MSCs can secrete a variety of cytokines that are involved in tissue damage repair (Additional file 1: Table S1). The inflammatory factors produced by the damaged sites can activate MSCs to secrete anti-inflammatory factors such as prostaglandin E2 (PGE2), IL-10, and TNF-α-induced protein 6 (TNAIP6 or TSG6). These factors inhibited the chemotaxis of neutrophils and macrophages, and maturation of dendritic cells, and reduced the proliferation of B and T lymphocytes [26–28]. Recent studies also reported that exosomes secreted by MSCs play a part in reducing myocardial ischemia/reperfusion injury [29], protecting acute kidney injury [30], regenerating nervous tissue [31], and reducing acute lung injury [32]. MSCs may inhibit the ability to repair damage and immunity adjustment by exosomes [33]. However, no matter which component secreted by MSCs takes part in tissue repair, our study confirmed that hucMSC-CM has the ability to protect against inflammation and renal fibrosis in vivo and in vitro. This finding is expected to optimize the drawbacks of stem cell transplantation, such as inconvenience, immunological rejection, and MSC tumorigenesis.

## Conclusions

Our results showed that hucMSC-CM reduced the inflammatory response via the TLR4/NF-κB signaling pathway and protected against UUO-induced CKD. The results of this study provide a new perspective on improving renal fibrosis by anti-inflammatory effects of hucMSC-CM. Further studies are needed to elucidate the active ingredients of hucMSC-CM as well as the underlying mechanisms that reduce renal inflammation, inhibit renal fibrosis, and improve CKD.

## Additional file

Additional file 1: Figure S1. Showing characterization of human umbilical cord mesenchymal stem cells (hucMSCs). **A** Flow cytometry analyses of phenotypic markers of hucMSCs; positive for CD73, CD90, and CD105, and negative for CD34, CD45, and HLA-DR. **B** Cell linage induced differentiation. Adipogenic differentiation analyzed by Oil Red O staining; after induction, hucMSCs formed numerous Oil-Red-O-positive lipid droplets. Osteogenic differentiation analyzed by Alizarin Red staining; after induction, hucMSCs formed Alizarin Red-positive mineral nodes. **Table S1** presenting cytokines and growth factor levels present in conditioned medium derived from hucMSC. Levels of insulin-like growth factor (IGF)-1, hepatocyte growth factor (HGF), stromal cell-derived factor (SDF)-1, brain-derived neurotrophic factor (BDNF), vascular cell adhesion protein (VCAM)-1, and transforming growth factor (TGF)-β in hucMSC-CM also measured using ELISA kits: human IGF-1 ELISA, human BDNF ELISA, human TGF-β ELISA (RayBiotech); and human CXCL12/SDF-1α quantikine ELISA kit, human HGF quantikine ELISA kit, human VCAM-1 quantikine ELISA kit (R&D Systems). Data expressed as mean ± SD. (DOCX 111 kb)

## Abbreviations

BUN: Blood urea nitrogen
CCTCC: China Center for Type Culture Collection
CKD: Chronic kidney disease
CM: Conditioned medium
DMEM/F12: Dulbecco’s modified Eagle’s medium nutrient mixture F-12
ECM: Excessive extracellular matrix
EMT: Epithelial–mesenchymal transition
ESRD: End-stage renal disease
HE: Hematoxylin–eosin
hucMSC: Human umbilical cord-derived mesenchymal stem cell
IOD: Integrated optical density
MCP-1: Monocyte chemotactic protein 1
PAS: Periodic acid–Schiff
PBS: Phosphate-buffered saline
PVDF: Polyvinylidene fluoride
SCr: Serum creatinine
SDS-PAGE: Sodium dodecyl sulfate polyacrylamide gel electrophoresis
TGF-β1: Transforming growth factor β1
UUO: Unilateral ureteral obstruction
