# Correction to: Human umbilical cord mesenchymal stem cell conditioned medium attenuates renal fibrosis by reducing inflammation and epithelial-to-mesenchymal transition via the TLR4/NF-κB signaling pathway in vivo and in vitro

**DOI:** 10.1186/s13287-018-0845-x

**Published:** 2018-03-22

**Authors:** Bo Liu, Fengxia Ding, Dong Hu, Yu Zhou, Chunlan Long, Lianju Shen, Yuanyuan Zhang, Deying Zhang, Guanghui Wei

**Affiliations:** 1 Department of Urology, Children’s Hospital of Chongqing Medical University, Chongqing, 400014 China; 2 Department of Respiratory Medicine, Children’s Hospital of Chongqing Medical University, No. 136, Zhongshan 2 RD, Yuzhong District, Chongqing, 400014 China; 3 Ministry of Education Key Laboratory of Child Development and Disorders, Chongqing Key Laboratory of Children Urogenital Development and Tissue Engineering, Chongqing Key Laboratory of Pediatrics, Chongqing International Science and Technology Cooperation Center for Child Development and Disorders, Chongqing, 400014 China; 4 Wake Forest Institute for Regenerative Medicine, Wake Forest School of Medicine, Winston-Salem, NC 27101 USA

## Erratum

The original article [1] contains an error whereby the corresponding authorship is mistakenly designated to the author Fengxia Ding.

The authors would like to clarify that Deying Zhang is the actual corresponding author for this manuscript, and all queries concerning the manuscript should be addressed to deyingzhang@126.com instead.
