# Supplementary material for: Human umbilical cord mesenchymal stem cell conditioned medium attenuates renal fibrosis by reducing inflammation and epithelial-to-mesenchymal transition via the TLR4/NF-κB signaling pathway in vivo and in vitro
Source: Stem Cell Res Ther. 2018 Jan 12;9:7. doi: 10.1186/s13287-017-0760-6 (PMC5767037; doi:10.1186/s13287-017-0760-6)
Supplement: Additional file 1: Figure S1. — Showing characterization of human umbilical cord mesenchymal stem cells (hucMSCs). A Flow cytometry analyses of phenotypic markers of hucMSCs; positive for CD73, CD90, and CD105, and negative for CD34, CD45, and HLA-DR. B Cell linage induced differentiation. Adipogenic differentiation analyzed by Oil Red O staining; after induction, hucMSCs formed numerous Oil-Red-O-positive lipid droplets. Osteogenic differentiation analyzed by Alizarin Red staining; after induction, hucMSCs formed Alizarin Red-positive mineral nodes. Table S1 presenting cytokines and growth factor levels present in conditioned medium derived from hucMSC. Levels of insulin-like growth factor (IGF)-1, hepatocyte growth factor (HGF), stromal cell-derived factor (SDF)-1, brain-derived neurotrophic factor (BDNF), vascular cell adhesion protein (VCAM)-1, and transforming growth factor (TGF)-β in hucMSC-CM also measured using ELISA kits: human IGF-1 ELISA, human BDNF ELISA, human TGF-β ELISA (RayBiotech); and human CXCL12/SDF-1α quantikine ELISA kit, human HGF quantikine ELISA kit, human VCAM-1 quantikine ELISA kit (R&D Systems). Data expressed as mean ± SD. (DOCX 111 kb) [file 13287_2017_760_MOESM1_ESM.docx]

**
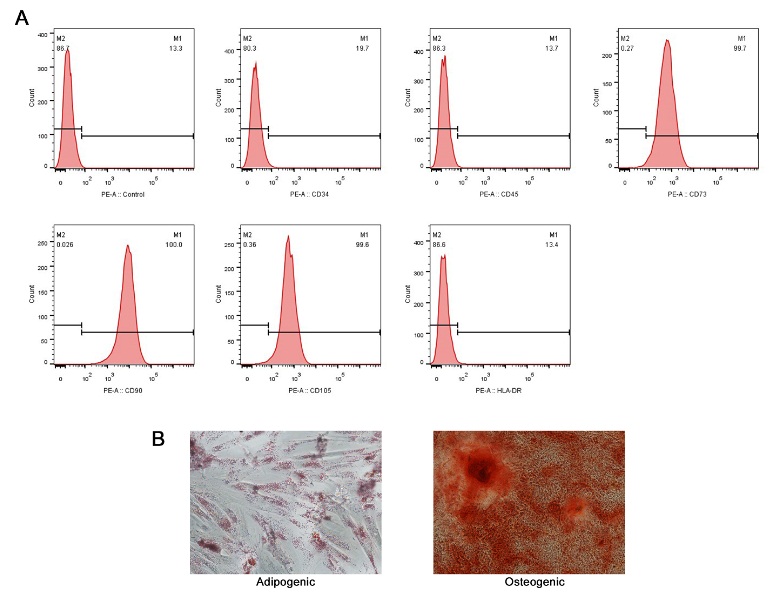
**

**Additional file 1: Figure S1. Characterization of human umbilical cord mesenchymal stem cells (hucMSCs).** (A) Flow cytometry analyses of phenotypic markers of hucMSCs. HucMSCs were positive for CD73, CD90, CD105, and negative for CD34, CD45 and HLA-DR. (B) Cell linage induced differentiation. Adipogenic differentiation was analyzed by oil red O staining. After induction, hucMSCs formed numerous oil-red-O-positive lipid droplets. Osteogenic differentiation was analyzed by alizarin red staining. After induction, hucMSCs formed alizarin red-positive mineral nodes.

**Additional file 1: Table S1．Cytokines and growth factor levels present in conditioned medium derived from hucMSC.**

| Cytokine | Assay | Conditioned medium  (n=3, pg/ml) |
| --- | --- | --- |
| BDNF  SDF-1 | ELISA  ELISA | 13949.50±2302.45  760.51±40.27 |
| IGF | ELISA | 859.90±73.91 |
| VCAM-1 | ELISA | 552.38±59.41 |
| TGF-β | ELISA | 4353.51±751.48 |
| HGF | ELISA | 654.36±29.46 |

The levels of insulin-like growth factor (IGF)-1, HGF, SDF-1, brain-derived neurotrophic factor (BDNF), vascular cell adhesion protein (VCAM)-1 and transforming growth factor (TGF)-β in hucMSC-CM were also measured using ELISA kits (Human IGF-1 ELISA, human BDNF ELISA, human TGF-β ELISA, RayBiotech; and human CXCL12/SDF-1α quantikine ELISA kit, human HGF quantikine ELISA kit, human VCAM-1 quantikine ELISA kit, R&D Systems). Data are expressed as the mean ± standard deviation. BDNF, brain-derived neurotrophic factor; SDF, stromal cell-derived factor; IGF, insulin-like growth factor; VCAM, vascular cell adhesion molecule; TGF, transforming f=growth factor; HGF, hepatocyte growth factor.
